# Behavioural Cardiology: A Review on an Expanding Field of Cardiology—Holistic Approach

**DOI:** 10.3390/jpm15080355

**Published:** 2025-08-04

**Authors:** Christos Fragoulis, Maria-Kalliopi Spanorriga, Irini Bega, Andreas Prentakis, Evangelia Kontogianni, Panagiotis-Anastasios Tsioufis, Myrto Palkopoulou, John Ntalakouras, Panagiotis Iliakis, Ioannis Leontsinis, Kyriakos Dimitriadis, Dimitris Polyzos, Christina Chrysochoou, Antonios Politis, Konstantinos Tsioufis

**Affiliations:** 1 First Department of Cardiology, National and Kapodistrian University of Athens, Hippokration Hospital, 114 Vassilissis Sofias Avenue, 11527 Athens, Greece; maria.spnor@gmail.com (M.-K.S.); begairini@gmail.com (I.B.); panos.tsioufis@gmail.com (P.-A.T.); myrto.pal@gmail.com (M.P.); ioantalak@gmail.com (J.N.); panayiotisiliakis@gmail.com (P.I.); giannisleontsinis@gmail.com (I.L.); dimitriadiskyr@yahoo.gr (K.D.); dim_polyzos@hotmail.com (D.P.); chrysohoou@usa.net (C.C.); ktsioufis@hippocratio.gr (K.T.); 2 First Department of Psychiatry, Eginition Hospital, Vas. Sofias 72–74, 11528 Athens, Greece; a_prentakis@yahoo.gr (A.P.); kontogianni.ev@gmail.com (E.K.); antnplts58@gmail.com (A.P.)

**Keywords:** behavioural cardiology, cardiovascular disease, psychosocial risk factors, metabolic factors, HEARTBEAT model, personalised medicine, digital health

## Abstract

Cardiovascular disease (CVD) remains Europe’s leading cause of mortality, responsible for >45% of deaths. Beyond established risk factors (hypertension, diabetes, dyslipidaemia, smoking, obesity), psychosocial elements—depression, anxiety, financial stress, personality traits, and trauma—significantly influence CVD development and progression. Behavioural Cardiology addresses this connection by systematically incorporating psychosocial factors into prevention and rehabilitation protocols. This review examines the HEARTBEAT model, developed by Greece’s first Behavioural Cardiology Unit, which aligns with current European guidelines. The model serves dual purposes: primary prevention (targeting at-risk individuals) and secondary prevention (treating established CVD patients). It is a personalised medicine approach that integrates psychosocial profiling with traditional risk assessment, utilising tailored evaluation tools, caregiver input, and multidisciplinary collaboration to address personality traits, emotional states, socioeconomic circumstances, and cultural contexts. The model emphasises three critical implementation aspects: (1) digital health integration, (2) cost-effectiveness analysis, and (3) healthcare system adaptability. Compared to international approaches, it highlights research gaps in psychosocial interventions and advocates for culturally sensitive adaptations, particularly in resource-limited settings. Special consideration is given to older populations requiring tailored care strategies. Ultimately, Behavioural Cardiology represents a transformative systems-based approach bridging psychology, lifestyle medicine, and cardiovascular treatment. This integration may prove pivotal for optimising chronic disease management through personalised interventions that address both biological and psychosocial determinants of cardiovascular health.

## 1. Introduction

**Figure 1:**
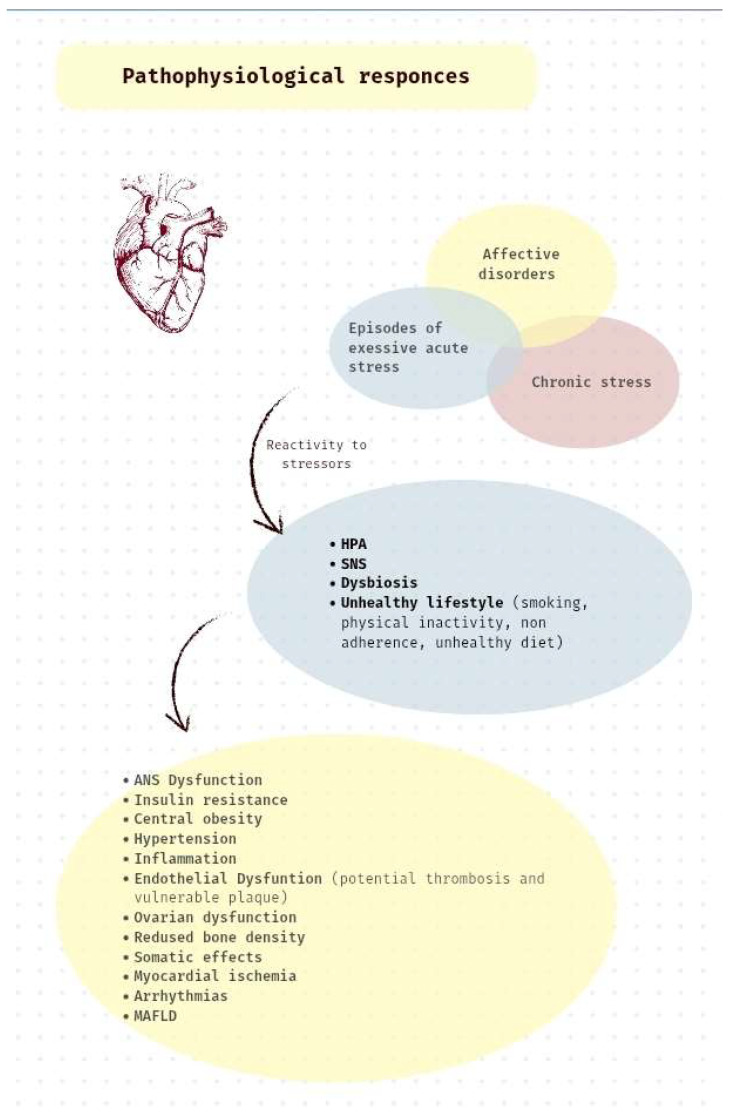
Psychological stress and disturbances leading to risk factors and cardiovascular disease. ANS: autonomic nervous system; HPA: hypothalamic–pituitary–adrenal; MAFLD: metabolic dysfunction–associated fatty liver disease; SNS: sympathetic nervous system. Episodes of excessive stress (illness), chronic cumulative stress (e.g., anxiety), and mood disorders (e.g., depression) provoke pathophysiological responses by activating the HPA axis and the SNS, while causing dysbiosis in patients with adopted unhealthy lifestyles and behaviours. A vicious circle of the aforementioned is born, regenerated by further external stressors (e.g., divorce, illness, death). The final chronic and acute outcomes of this pathophysiological pathway involve established risk factors for cardiovascular disease (such as insulin resistance, central obesity, MAFLD) and cardiovascular events (such as myocardial ischemia and arrhythmias). Dysbiosis is referred to as gut dysbiosis. Somatic effects include symptoms such as abdominal pain, dyspepsia, chest pain, fatigue, dizziness, insomnia, and headache. Modified by Rozanski et al. [5].

Cardiovascular disease (CVD) is the leading cause of illness and death globally, resulting in around 3.9 million fatalities each year in Europe, which accounts for 45% of all deaths [1]. The financial burden is equally significant, with the European Union incurring costs exceeding EUR 210 billion annually due to direct healthcare expenses, productivity losses, and long-term disabilities [2]. Historically, efforts in cardiovascular prevention and rehabilitation have concentrated on modifiable biological risk factors like hypertension, diabetes, high cholesterol, smoking, lack of physical activity, and obesity. While these components continue to be vital, recent evidence demonstrates that psychosocial factors also play a crucial role in the onset and progression of CVD [3,4,5] (Figure 1).

**Figure 2:**
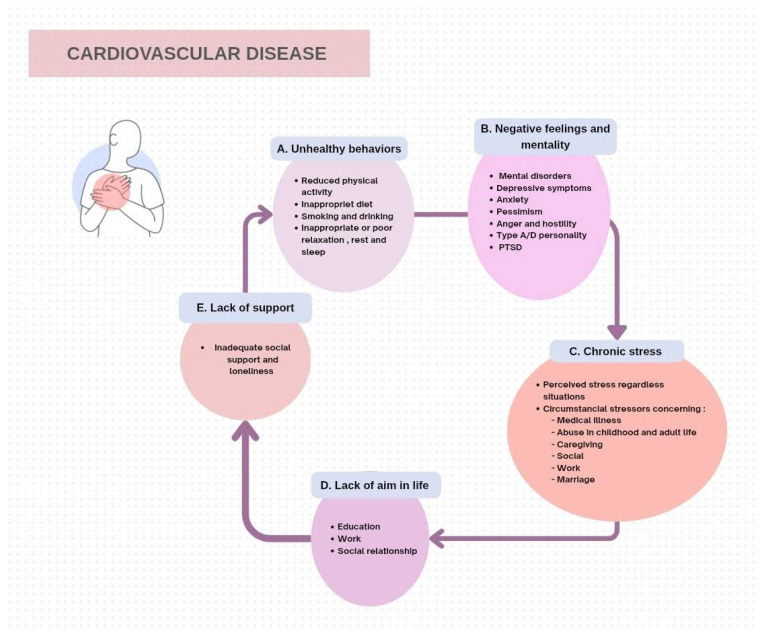
Stressors leading to cardiovascular disease. PTSD: Post-Traumatic Stress Disorder. Different types of stressors that could provoke cardiovascular disease.

This shift in understanding has fostered the emergence of Behavioural Cardiology, an integrative field that incorporates psychological, social, and behavioural elements into the evaluation and management of cardiovascular disorders [6]. In particular, mental health issues such as depression and anxiety, negative personality traits (including hostility and the Type A behavioural pattern), socioeconomic challenges, past traumatic events, and one’s perception of stress are linked to a higher risk of heart attacks, arrhythmias, and cardiac-related deaths [7,8,9] (Figure 2). Despite their high prevalence, these issues frequently go overlooked and untreated in cardiology practices. A comprehensive meta-analysis by Su et al. [10] affirmed that psychological eHealth interventions significantly reduce depressive symptoms in CVD patients (SMD ≈ −0.30), underscoring their potential to improve psychosocial and cardiovascular outcomes. Moreover, Moazzami et al. [11] introduced a novel cardiovascular reactivity risk score derived from hemodynamic and endothelial responses during acute stress tasks; individuals in the highest quartile showed nearly double the risk of future adverse cardiac events over six years. Finally, Fuhrmann et al. [12] conducted an 18-day RCT of the MT-StressLess smartphone app with integrated heart-rate biofeedback, demonstrating a moderate effect size (d = 0.41) on stress reduction—highlighting scalable digital tools as viable complements in cardiac stress management.

**Figure 3:**
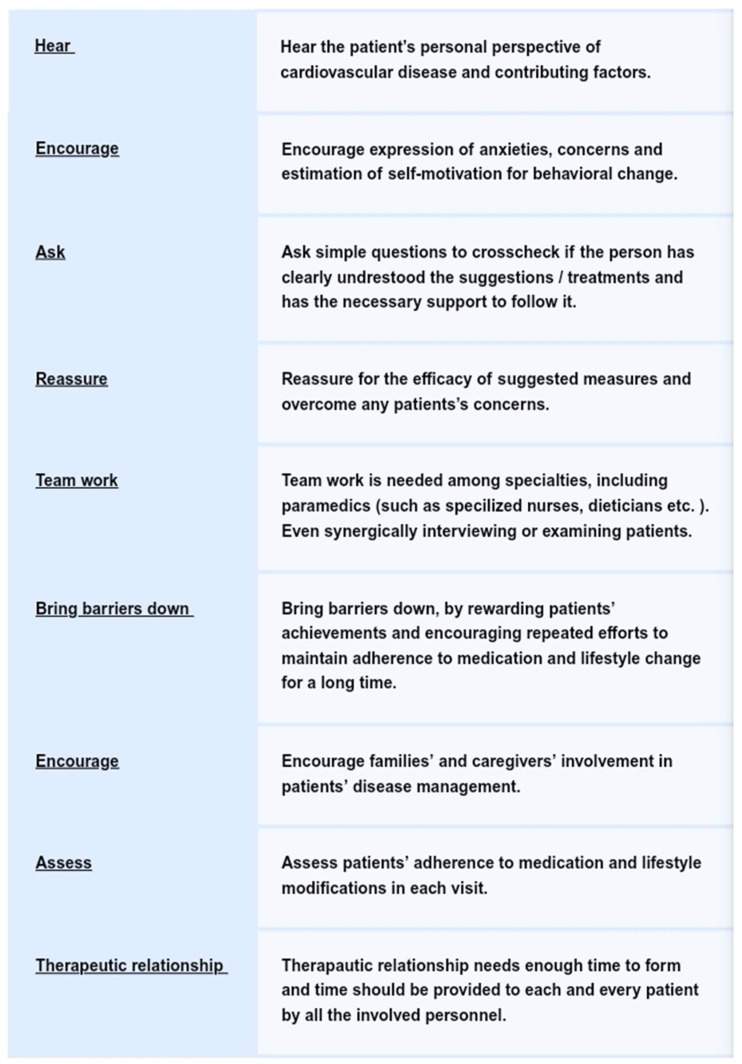
HEARTBEAT approach of patients in the Behavioural Cardiology Unit in a Greek Hospital. The first letter of the first word of each measure forms the acronym HEARTBEAT.

The HEARTBEAT model, created by the Behavioural Cardiology Unit at a tertiary care hospital in Greece, provides a structured approach to addressing these psychosocial factors in both primary and secondary prevention contexts (Figure 3). Primary prevention focuses on individuals at heightened risk of CVD who have not yet experienced a clinical cardiovascular event, while secondary prevention aims at patients with existing disease, emphasising the prevention of recurrence and complications [13].

Importantly, Behavioural Cardiology and the HEARTBEAT model advocate for a personalised medical approach by customising interventions according to individual psychosocial and behavioural traits. This customisation is achieved through validated assessment instruments, personality and stress evaluations, emotional screenings, caregiver involvement, and assessments of health literacy [14]. The HEARTBEAT method facilitates tailored strategies that consider patient preferences, psychological resilience, support systems, and cultural contexts, providing an alternative to traditional, algorithm-driven cardiac care. Increasingly, personalised digital tools, such as mobile health (mHealth) applications and telemonitoring, are becoming fundamental to this framework, enhancing patient engagement, adherence, and health results [15]. Furthermore, this review highlights successful international initiatives, including the German Cardiac Society’s 2018 position statement on psychosocial factors [3], the UK’s UPBEAT study [16], and new efforts in Scandinavia and the Netherlands that combine digital resources with behavioural strategies [17]. By positioning the Greek HEARTBEAT model within this international context, this review underscores its innovative potential and the opportunity to expand such systems. Finally, it is important to clarify that this review emphasises Behavioural Cardiology as a discipline while using the HEARTBEAT model as a practical example. Therefore, it aims to establish a framework for evidence-based, psychosocially informed cardiovascular care that can be adapted for diverse cultural and healthcare environments through a personalised, patient-centred approach.

A literature review was performed to explore how Behavioural Cardiology establishes a basis for a personalised medicine approach by incorporating individual psychosocial profiles into risk evaluation and treatment strategies. The search was conducted on Medline using keywords such as Behavioural Cardiology, cardiovascular disease, psychosocial risk factors, prevention, personalised medicine, digital health, and telemedicine, utilising the Boolean operators AND and OR. Only articles written in English from 1990 onward were considered, with the latest search taking place in June 2025.

## 2. Psychosocial Factors and Coronary Artery Disease

Psychosocial risk elements—such as chronic stress, depression, social isolation, and certain personality characteristics—are acknowledged as significant factors in the onset and progression of coronary artery disease (CAD). These factors affect cardiovascular health through various pathways, including the alteration of the hypothalamic–pituitary–adrenal (HPA) axis, heightened levels of inflammatory cytokines, excessive stimulation of the sympathetic nervous system, and impaired endothelial function [3,8,9]. The European Society of Cardiology (ESC) officially acknowledges psychosocial factors as independent influencers of cardiovascular risk [13]. Research shows that encountering several psychosocial stressors can collectively increase the risk of developing CAD. For instance, the INTERHEART study found that individuals enduring chronic stress are more than twice as likely to experience a myocardial infarction compared to those not experiencing such stress [18]. A systematic review by Gaffey et al. [4] validated that psychological distress significantly heightens the risk of new cardiovascular disease cases and related mortality. The psychosocial domains highlighted in Behavioural Cardiology—including mood disorders (like depression and dysthymia), anxiety, trauma-related disorders, and personality traits such as hostility—are supported by both ESC guidelines and extensive meta-analyses [19,20]. Furthermore, the German Cardiac Society noted in its 2018 update that persistent emotional stress and mental health challenges are not merely comorbidities but also play an active role in the development of atherosclerosis and cardiac incidents [3]. While the term “dose-dependent” is occasionally used to describe the relationship between psychological stress and the severity of cardiovascular illness, it should be interpreted qualitatively rather than in a pharmacokinetic context. Prolonged exposure to stressors like loneliness or work-related pressures has been shown to worsen endothelial dysfunction and increase C-reactive protein levels, which are both indicators of CAD [7,8]. Nonetheless, there remain gaps in our understanding. Numerous psychosocial factors—such as loneliness, grief, and post-traumatic stress—have not been adequately examined regarding their mechanistic pathways and potential therapeutic approaches. Further investigation is essential to clarify causal relationships and develop focused treatment strategies.

## 3. Work and Home Stress

Occupational and domestic stress are among the most commonly mentioned psychosocial factors associated with hypertension and heart disease. Elevated job strain, characterised by high demands coupled with limited control, has been linked to a rise in the risk of coronary events by as much as 23% [21]. A more recent comprehensive review of 18 studies involving 232,767 participants found that high work-related psychosocial stress raises the risk of cardiovascular events (including coronary heart disease and myocardial infarction) by ~26% (HR = 1.26; 95% CI 1.12–1.41) [22]. The Cornell Worksite Blood Pressure Project, a cohort study, notably found that individuals experiencing high job strain exhibited a substantially increased left ventricular mass index and workplace diastolic blood pressure [23]. Unlike earlier misclassifications, this study incorporated prospective elements and was not solely case–control.

Stress encountered at home, including caregiving challenges, relationship issues, and financial strain, may activate the sympathetic nervous system, leading to poor sleep quality and lower adherence to medical treatment. The PURE study [24], which involved 17 countries, uncovered a significant link between psychosocial stress and obesity, hypertension, and new cases of cardiovascular disease. This conclusion has been supported by more recent research by Santosa et al. [7], who reviewed data from the same cohort using advanced methodologies.

In studying the cardiovascular impacts of stress, hypertension serves as a more pertinent and clinically significant outcome than merely an increase in “blood pressure.” Continuous exposure to stress is recognised to raise both resting systolic and diastolic pressures through imbalanced autonomic function and disturbed nocturnal blood pressure dipping [25].

A recent review by Vaccarino and Bremner [9] confirmed that the cardiovascular responses triggered by stress—particularly in the setting of low social support—are strong indicators of negative outcomes, including heart failure and sudden cardiac death. These findings underscore the necessity to consider both workplace and home-related psychosocial stressors when assessing cardiovascular risk. However, current interventions are inadequately developed. Future research should concentrate on integrating stress-reduction strategies into individualised prevention programs, which may encompass mindfulness-based cognitive therapy and mobile applications for stress tracking, as part of a comprehensive Behavioural Cardiology approach.

## 4. Pessimism Versus Optimism

Dispositional optimism, characterised as the anticipation of favourable outcomes, is gaining recognition as a protective factor against cardiovascular events, while pessimism correlates with elevated rates of morbidity and mortality [14,26]. These characteristics influence cardiovascular health through behavioural, autonomic, and inflammatory pathways, affecting biological responses to stress, treatment compliance, and lifestyle decisions [9,27]. A meta-analysis conducted in 2019, which included over 200,000 participants, indicated that optimism was associated with a 35% lower risk of CVD events and mortality overall [26]. Subsequent studies have built on these results. Whitfield et al. [27] showed that pessimism, as opposed to optimism, was independently linked to an elevated risk of cardiovascular death among 29,000 Australians. In a similar vein, Krittanawong et al. [28] and Krittanawong et al. [29] discovered that higher levels of pessimism were indicators of adverse cardiovascular outcomes, such as myocardial infarction, stroke, and sudden cardiac death, while optimism was associated with enhanced endothelial function and decreased inflammatory markers.

The incorporation of personality-centred risk assessments in Behavioural Cardiology marks a meaningful advancement in personalised medicine. The HEARTBEAT model integrates recognised psychological assessments, including the Life Orientation Test—Revised (LOT-R), into routine clinical evaluations to pinpoint individuals at heightened psychological risk. Customised cognitive behavioural therapy (CBT), resilience training, and health coaching are utilised to tackle maladaptive perspectives [30]. These resources enable healthcare providers to classify patients for preventive measures based on their emotional style and stress vulnerability, aligning with the principles of individualised care.

Despite the strong evidence backing these findings, the ideas of pessimism and optimism are still infrequently employed in clinical cardiology. Future investigations should assess how the inclusion of these traits in electronic health records and risk assessment tools could enhance prognostic precision and the implementation of interventions across various contexts.

## 5. Depression

Depression is recognised as a major psychosocial risk factor related to cardiovascular diseases, with extensive studies emphasising its significance. It affects nearly 20% of individuals with CAD and is linked to a doubled risk of cardiovascular events and mortality [19,31]. Mechanistically, depression promotes inflammation, autonomic dysfunction, platelet aggregation, and poor adherence to health-promoting behaviours, all of which contribute to disease onset and impede recovery efforts [4]. Longitudinal results from the UPBEAT study conducted in the UK reveal that depression can occur both before and after cardiovascular events, establishing a cycle of heightened vulnerability [16]. It is particularly noteworthy that women and younger individuals appear to be at increased risk, potentially due to hormonal factors and differences in emotional processing [32].

The HEARTBEAT model incorporates depression screenings using standardised tools like the Hospital Anxiety and Depression Scale (HADS) and the Depression, Anxiety and Stress Scales (DASS-21). For those identified as high risk, interdisciplinary interventions are launched, which may include medication, CBT, and enhancing social support. This holistic approach exemplifies personalised medicine, tailoring the type and intensity of treatment based on each person’s symptoms, cognitive styles, and available support networks.

Importantly, while depression is commonly addressed within Behavioural Cardiology, there is a lack of research into how it interacts with other psychosocial factors such as pessimism, anxiety, or trauma. Tackling this gap through comprehensive psychological assessments may result in more effective and integrated care strategies.

## 6. Post-Traumatic Stress Disorder

Post-Traumatic Stress Disorder (PTSD) is increasingly acknowledged as a crucial psychological influence on CVD, especially among those with prior trauma, veterans, and individuals recovering from acute coronary incidents. From a mechanistic perspective, PTSD leads to heightened sympathetic nervous system activity, endothelial dysfunction, systemic inflammation, and an increased propensity for blood clot formation, all of which contribute to the development of atherosclerosis and enhanced cardiac vulnerability [33,34]. Research has shown that veterans who have PTSD demonstrate reduced brachial artery flow-mediated dilation and increased carotid intima-media thickness—both indicators of early endothelial damage [33]. The MI-SPRINT study revealed that implementing early trauma-focused therapy after a myocardial infarction significantly mitigated PTSD symptoms and improved patient outcomes [35]. In addition to vascular implications, PTSD triggers behavioural shifts that heighten the risk of CVD, including poor adherence to medication, substance misuse, sleep disturbances, and social isolation. Recent studies also highlight the influence of neuroendocrine dysfunction and genetic factors in linking PTSD to cardiovascular diseases [34,36].

The HEARTBEAT model emphasises the importance of acknowledging trauma backgrounds and PTSD symptoms as critical components of its comprehensive assessment process. Individuals suspected of having PTSD are referred for psycho-diagnostic assessment and provided with evidence-based interventions, such as CBT and trauma-focused counselling [30]. Moreover, lifestyle changes, including adherence to the Mediterranean diet and encouragement of physical activity, have been shown to effectively reduce cardiovascular risks in those with PTSD [36].

Despite these hopeful approaches, PTSD is often overlooked in cardiology settings. Enhancing screening protocols, particularly for women, minority populations, and patients who have experienced intensive care unit (ICU) stays, is vital. These groups often face unique barriers, including cultural stigma, lack of mental health literacy, and limited access to trauma-informed care. For example, women are more likely to internalise trauma symptoms as somatic complaints—such as fatigue, sleep disturbance, or palpitations—which can be mistaken for non-psychiatric issues, especially in cardiology clinics. Older adults and migrant populations may also underreport traumatic experiences due to shame, generational differences, or language barriers [37]. This under-reporting delays recognition and treatment, leaving cardiovascular risk factors unaddressed. To counter this, the HEARTBEAT model encourages the use of structured trauma screening tools and interdisciplinary dialogue to ensure that trauma-related symptoms are neither overlooked nor misattributed to unrelated causes. Future investigations should focus on developing scalable digital tools and genetic markers to enable early identification and personalised treatment within the realm of Behavioural Cardiology.

## 7. Phobic Anxiety

Phobic anxiety is an important yet frequently overlooked element that contributes to CVD. It refers to enduring and irrational fears that result in autonomic arousal and behavioural avoidance, both of which can adversely affect cardiovascular well-being. Initial research has suggested that phobic anxiety could be a predictor of sudden cardiac death, especially among women [38,39].

Recent studies further reinforce this connection. A 2016 meta-analysis conducted by Emdin et al. found that anxiety, including its phobic forms, was linked to a 26% increased chance of developing coronary heart disease and a 48% increased risk of stroke [40]. Similarly, Batelaan et al. [41] discovered that individuals suffering from anxiety disorders faced a greater likelihood of experiencing new cardiovascular events, regardless of lifestyle and metabolic factors.

The relationship between phobic anxiety and cardiovascular disease is associated with sustained rises in heart rate and blood pressure, enhanced platelet activity, and the dysfunction of the HPA axis. Furthermore, the inclination to avoid seeking medical assistance, poor adherence to medications, and ineffective coping mechanisms may further elevate cardiovascular risk.

The HEARTBEAT model includes systematic anxiety screenings using tools like the HADS scale, followed by personalised interventions such as CBT or exposure therapy. Healthcare providers are encouraged to tackle not only the anxiety symptoms but also the resulting behaviours, such as neglecting medical check-ups or rehabilitation sessions.

Considering the chronic and often unaddressed aspect of phobic anxiety, particularly in older adults and those with low health literacy, regular identification and focused treatment could be essential for effectively preventing cardiovascular diseases. Future studies should explore how digital exposure therapy and wearable physiological monitors can improve personalised approaches to managing anxiety.

## 8. Anger and Hostility

Research indicates that emotions such as anger and hostility, along with characteristics linked to the “Type A” behaviour pattern, have been historically associated with a higher likelihood of cardiovascular issues. While the Type A personality was initially defined by traits like competitiveness, urgency concerning time, and aggressiveness, it is now more accurately connected to hostility—a constant characteristic characterised by cynicism, distrust, and a tendency toward anger. These emotional states may lead to negative cardiac outcomes through several mechanisms, including increased sympathetic activity, endothelial dysfunction, oxidative stress, and elevated inflammatory markers [17,19].

Recent studies have improved our understanding of how moments of anger impact vascular health. In a randomised controlled study, Shimbo et al. [17] found that short bursts of anger provocation significantly reduced flow-mediated dilation, indicating an immediate negative effect on endothelial function. Additionally, Titova et al. [42], in a study involving 47,000 adults from Sweden, revealed that frequent anger episodes were independently linked to heart failure and cardiovascular mortality, particularly among men and individuals with diabetes.

Despite this strong correlation, anger tends to be overlooked in clinical cardiology. The HEARTBEAT model underscores the importance of identifying anger through validated tools like the Cook–Medley Hostility Scale and structured interviews. Patients exhibiting high anger reactivity are provided with targeted interventions such as anger management training, mindfulness-based stress reduction (MBSR), and cognitive restructuring. These methods are tailored to fit the individual’s emotional characteristics and stress triggers, highlighting the model’s commitment to personalised behavioural medicine. Moreover, interdisciplinary care teams consisting of psychologists, cardiologists, and health coaches are trained to identify behavioural signs of hostility and incorporate these factors into long-term risk reduction strategies. Consequently, addressing anger becomes a dual psychological and physiological goal within cardiovascular care (Figure 1).

## 9. Socioeconomic Status

Socioeconomic status (SES) is a significant factor in cardiovascular health, influencing access to healthcare, adherence to medications, understanding of health information, nutrition, and levels of stress. Individuals with lower SES consistently exhibit higher rates of CVD, poorer health outcomes, and reduced use of preventive healthcare services [43,44]. The PURE study [44] highlighted how disparities related to SES affect the incidence and management of major cardiovascular risk factors across 21 countries. Additionally, Walli-Attaei et al. [32] showed that low-income women encounter specific challenges, facing significant barriers to accessing statins, cardiac rehabilitation, and specialised care, which exacerbates health disparities based on gender.

In Greece, the socioeconomic crisis resulting from the financial downturn and subsequent austerity measures worsened healthcare inequalities. There was a reduction in funding for cardiac rehabilitation programs, and preventive services were deprioritised. The HEARTBEAT model was developed partly to address these systemic issues. Its objective is to reduce disparities by implementing universal screening for psychosocial risk factors, providing flexible service delivery methods (such as telehealth), and enabling referrals to social work for patients experiencing financial difficulties.

Importantly, the HEARTBEAT model evaluates SES not only through income but also by taking into account education, housing conditions, social support, and experiences of discrimination. This comprehensive assessment facilitates the customisation of interventions, such as offering nutrition counselling tailored for individuals facing food insecurity or mobile application assistance for patients with limited access to healthcare services.

The COVID-19 pandemic has intensified SES-related disparities, especially in low-resource settings. Vulnerable populations have faced higher rates of infection, delays in cardiac care, and increased psychosocial stress. To tackle these emerging issues, Behavioural Cardiology must evolve by promoting digital health equity, creating adaptable care pathways, and establishing community-based intervention hubs to support at-risk groups.

## 10. The Elderly

As global populations age, cardiovascular care must increasingly address the complex needs of individuals in advanced age. Although age 65 is commonly used as a clinical threshold for defining “older adults,” this marker is largely based on social conventions such as retirement, rather than biological aging [45,46,47,48]. Older adults represent a highly heterogeneous group in terms of functional capacity, comorbid conditions, and psychosocial vulnerability, all of which affect CVD risk and outcomes. While frailty—a syndrome characterised by reduced physiological reserve and increased vulnerability to stressors—has long been a focal point in geriatric cardiology, it is only one piece of a broader spectrum of age-related concerns. Geriatric syndromes such as cognitive impairment, polypharmacy, depressive symptoms, mobility limitations, and nutritional deficiencies are also critical determinants of cardiovascular risk and recovery [45,46,47].

The HEARTBEAT model explicitly addresses these multidimensional aspects of aging. Comprehensive geriatric assessment (CGA) is integrated into its intake process to capture both clinical and functional domains. Cognitive tools such as the Mini-Cog [49] and the Montreal Cognitive Assessment (MoCA) [50], frailty indices (e.g., Clinical Frailty Scale) [51], and mobility assessments (e.g., Timed Up and Go Test) [52] are integrated into routine practice. Based on these assessments, individualised care plans are developed, often involving multidisciplinary collaboration across geriatrics, cardiology, psychology, and physiotherapy.

Importantly, older adults often face systemic barriers to care—including limited transportation, social isolation, digital exclusion, and caregiver burden—that are not adequately addressed in traditional cardiology models. The HEARTBEAT framework incorporates caregiver engagement, community outreach, and telehealth modalities to provide flexible, personalised care adapted to the unique circumstances of each patient. Culturally tailored approaches are also necessary, as aging is experienced differently across societies. For instance, attitudes toward autonomy, family caregiving, and end-of-life preferences vary widely and must be considered when designing behavioural interventions for older adults. Ultimately, integrating geriatric principles into cardiovascular prevention and treatment promotes more equitable, individualised care and reduces the risk of overtreatment or neglect in this growing patient group. Future work should focus on developing scalable models that combine Behavioural Cardiology with geriatric expertise to optimise outcomes for older adults with or at risk for CVD.

## 11. Assessing and Measuring

This review has established that various psychosocial factors—including depression, anxiety, chronic stress, and trauma—are strongly associated with the development and progression of CVD. However, the exact biological mechanisms through which these psychological states affect cardiovascular health remain incompletely defined, and it is still uncertain whether these associations follow a true dose–response pattern [53]. Current evidence shows that negative psychological states disrupt autonomic nervous system regulation, heighten cardiovascular reactivity, and contribute to metabolic changes such as central obesity and insulin resistance. They also adversely impact neurocognitive function, increase the risk of hypertension, and impair endothelial and platelet function, thereby accelerating atherosclerosis [19,53]. Chronic stress and depression, in particular, promote sympathetic overactivity, inflammation, and vascular dysfunction, all of which elevate the risk for adverse cardiac events [54]. Despite these established links, there is limited clarity regarding which specific stressors activate which pathophysiological pathways. This represents a key gap in the translation of psychosocial science into personalised cardiac care.

To address this, Behavioural Cardiology increasingly relies on standardised clinical tools to assess and quantify psychosocial risks in daily practice. The HEARTBEAT model incorporates several such instruments into its routine evaluation process. For instance, the UPBEAT study by Walters et al. [16] utilised the EuroQol 5 Dimension (EQ-5D) to assess health-related quality of life, the Social Problem-Solving Questionnaire (SPQ) to evaluate cognitive patterns, and the HADS to screen for mood disorders. These tools are particularly relevant in cardiovascular populations due to their brevity, ease of administration, and sensitivity to subclinical mood disturbances.

Similarly, Fezza et al. [55] and Nolan et al. [15] used the Kansas City Cardiomyopathy Questionnaire-05 (KCCQ-05) to measure quality of life in patients with heart failure. Nolan’s team also employed the Generalized Anxiety Disorder-7 (GAD-7), the Patient Health Questionnaire-9 (PHQ-9), and the 10-item Perceived Stress Scale (PSS-10) to identify psychological burden. In addition, they assessed patients’ readiness to adopt behavioural change using Prochaska and DiClemente’s transtheoretical model, which allows clinicians to tailor interventions to patients’ motivational stages [56].

Qualitative approaches are equally important. Open-ended questions such as “How would you describe your energy levels?”, “How have you been sleeping?”, and “Do you feel under pressure at work or home?” serve as effective entry points for identifying distress in clinical conversations [57]. These methods align with HEARTBEAT’s emphasis on person-centred, empathetic care.

In older adults, frailty and functional status are essential to evaluate when making cardiovascular treatment decisions. Tools such as the Clinical Frailty Scale, the FRAIL score, and the Green Score are increasingly used in both hospital and outpatient settings to identify vulnerable individuals [55,58]. These measures are central to risk stratification, care planning, and the prevention of treatment-related harm.

Despite the availability of these tools, further work is needed to determine which are most predictive, efficient, and scalable for use across diverse populations. Moreover, integration of psychosocial assessment into electronic health records and digital monitoring platforms remains limited but is essential for the future of personalised Behavioural Cardiology. There is still a need to develop an integrated scoring system to prioritise interventions in each patient. Also, dedicated nursing personnel, by fully assessing the patients using the aforementioned tools, could help overcome the clinical burden posed to the team’s doctors.

## 12. Comorbidity in Psychiatric Conditions

Mental health challenges like depression, anxiety, and post-traumatic stress do not always appear in isolation—they often show up together, interacting in ways that make life, and recovery, much harder. This overlap, known as psychiatric comorbidity, is a crucial but often overlooked aspect of Behavioural Cardiology. When conditions like depression and anxiety occur together, they tend to amplify the body’s stress response, disrupt sleep and motivation, and make it more difficult for patients to stick to medication or lifestyle changes. The combined emotional burden can also lead to increased inflammation and autonomic imbalance, further straining the heart. A recent meta-analysis by Su et al. [10] found that psychological eHealth tools work best when they address both depression and anxiety at the same time, rather than in isolation. This reinforces the need for transdiagnostic care models that recognise how closely these conditions are intertwined.

In practice, the HEARTBEAT model already includes screening tools like the HADS, DASS-21, and GAD-7 to help detect overlapping mental health concerns early. This allows clinicians to better tailor interventions—whether that means a stepped approach to cognitive behavioural therapy, supportive medication, or emotional support through counselling. Still, there is room to grow. Future research should dive deeper into how these psychiatric patterns interact—not just emotionally, but physiologically—in people with heart disease. Understanding these layers could help shape more compassionate, precise, and effective care strategies for patients facing both emotional and cardiovascular challenges.

## 13. Prevention

Preventing CVD today requires more than managing biological risk factors—it demands a holistic approach that also addresses psychological, behavioural, and social determinants of health. Behavioural Cardiology offers a framework that integrates these dimensions, and the HEARTBEAT model is a prime example of this approach in practice. Developed in Greece to address gaps in cardiac rehabilitation and preventive care, HEARTBEAT is a comprehensive, patient-centred model applicable to both primary and secondary prevention strategies.

The acronym HEARTBEAT represents nine interrelated components (Figure 3):

- Holistic evaluation;
- Engagement of caregivers and social support;
- Assessment using validated tools;
- Reinforcement of healthy behaviours;
- Team-based interdisciplinary care;
- Behavioural interventions;
- Education and empowerment;
- Adherence tracking;
- Technology-enabled monitoring.

In primary prevention, the model targets individuals at high risk for CVD—such as those with psychosocial stress, sedentary lifestyles, or poor dietary habits—before clinical symptoms emerge. Interventions focus on behaviour modification, emotional resilience, and early identification of psychological stressors. In secondary prevention, the HEARTBEAT model supports patients with existing CVD by integrating psychosocial support into rehabilitation and long-term disease management.

A tiered (or “stepped”) model for behavioural health care is proposed:

(a) In the first tier, general cardiologists are responsible for triage of patients with CVD or high risk for behavioural risk and provision of brief counselling.
(b) The second tier would involve physician referral of patients to behavioural intervention programs designed to provide integrated intervention across the wide domains of behavioural factors (diet, exercise, sleep hygiene, rest, relaxation, stress management, and time management practices). Why? Who? What measures fit?
(c) The third tier would involve the referral of patients and their families to behavioural specialists when depression, anxiety, stress, or other psychosocial issues are mandated. In this step, other subspecialties and specialties should be involved according to the needs of patients.

The following-up involves more frequent visits in the first three months (every 2 weeks if necessary) and occasional monitoring afterwards in the long term with close telephone monitoring by Behavioural Cardiology nurses.

Each element of the model is applied through structured tools and personalised strategies. For example, screening tools like the HADS, KCCQ-05, and Clinical Frailty Scale are used to assess mood disorders, quality of life, and physical vulnerability [16,55]. The Prochaska stages-of-change model helps tailor interventions to patients’ readiness for behaviour change [56], while caregiver involvement supports sustained adherence. Also, the HEARTBEAT model, through continuous follow-up and reassessment, detects patients’ psychosocial status variability over time, especially for factors such as pessimism or perceived stress that are highly dependent on life events. Through constant feedback, the medical care could adapt to patients’ real-time needs.

The HEARTBEAT framework is aligned with recent ESC guidelines, which emphasize the inclusion of mental health screening, social support, and behaviour-focused interventions in CVD prevention [13]. It also shares similarities with international efforts, such as the UPBEAT program in the UK and psychosocial cardiology initiatives in Germany [3]. These comparisons validate its relevance beyond the Greek healthcare system.

While the model originated in response to systemic limitations in Greece—such as a lack of structured rehabilitation programs—it is designed to be adaptable across healthcare settings. For instance, in lower-resource or rural environments, mobile health technologies, community networks, and remote consultation can be used to deliver their core components effectively. Furthermore, illiteracy, lack of social support, and lack of access to medical care are real-world problems, especially in developing and underdeveloped countries. Also, religion can influence eating habits and the patients’ engagement in disease self-management. These barriers could be overcome by encouraging caregivers’ involvement in disease management to overcome patients’ inability, as well as constant direct access to medical care. In addition, personalised lifestyle modifications could follow real personal needs according to religion or social beliefs.

### 13.1. Cost and Sustainability Considerations

Implementing a Behavioural Cardiology Unit involves added investment in time, personnel, and interdisciplinary coordination. However, the HEARTBEAT model could reduce hospital readmissions, improve medication adherence, and enhance patient satisfaction—awaiting the preliminary data of follow-up of this approach. In general, Behavioural Cardiology could serve as the much sought-after holistic approach that could engage even the younger patients as described by Rozanksi et al. [59]. Gaffey et al. [4] also report that systematic psychological screening paired with targeted behavioural therapy contributes to long-term cost savings through improved cardiovascular outcomes.

While the HEARTBEAT model presents a compelling holistic approach, the financial and logistical feasibility of its implementation within public health systems deserves closer scrutiny. Integrating psychologists, behavioural nurses, and digital health platforms requires upfront investment—not only in personnel and training, but also in infrastructure and ongoing coordination. Many national healthcare systems, especially in lower-income regions, already face staffing shortages, limited mental health integration, and tight budgets. This raises concerns about whether the proposed tiered care pathways and long-term follow-up schedules can be realistically scaled. However, formal economic evaluations specific to the HEARTBEAT model are still lacking. Public implementation may require stepwise adoption—starting with digital screening tools, short-term counselling pilots, and primary care-based interventions—before full rollout becomes viable.

### 13.2. Integrating Technology and Personalisation

Personalised prevention is further enhanced by incorporating digital tools. Remote monitoring devices, wearable technologies, and app-based platforms enable real-time tracking of stress levels, activity, and sleep, and facilitate patient engagement between visits [15]. These technologies also support individualised feedback and behavioural prompts based on patient-reported outcomes and physiological data.

**Figure 4:**
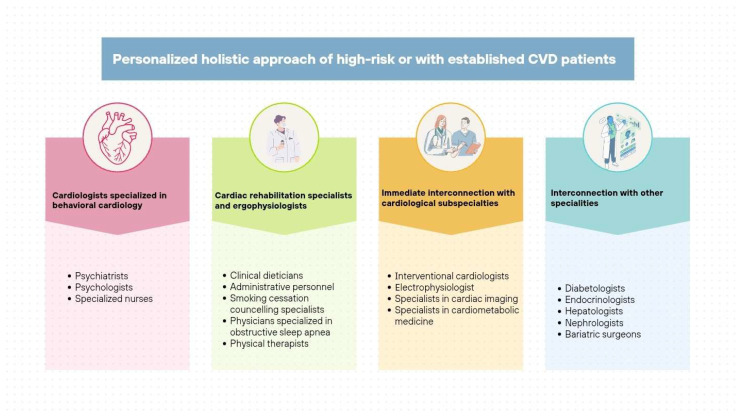
Personalised holistic management of patients with cardiovascular disease or high-risk factors by the Behavioural Cardiology Unit. CVD: Cardiovascular disease. The core of the Behavioural Cardiology Unit consists of cardiologists, psychiatrists, psychologists, and nurses specialised in this field. The unit should be assisted by cardiac rehabilitation specialists, dieticians, etc., who provide a holistic approach to patients with cardiovascular disease or high-risk factors. The overall team needs further interconnection with other cardiological subspecialties and other specialities to achieve high-quality management of each and every one of these patients that present many comorbidities, as well as their families and caregivers.

In this way, the HEARTBEAT model brings together psychosocial screening, behavioural science, digital health, and collaborative care into a flexible, forward-looking framework that meets the evolving needs of modern cardiovascular prevention (Figure 4).

### 13.3. Interprofessional Communication and Care Continuity

Strong collaboration between specialists, primary care doctors, and allied health teams is vital—yet often undervalued—when caring for patients with multiple complex conditions. While systems like the ‘Hub and Spoke’ model improve efficiency, they can unintentionally create challenges. Patients may face inconvenient logistics, and care can feel disconnected as it is split across different providers.

The model works like this: A central ‘Hub’ hospital handles specialised, complex care, while local ‘Spoke’ clinics manage everyday health needs. This approach helps match patients with the right level of care, making smart use of resources while aiming for better health outcomes. But the real key to success? Seamless communication—both between different care levels and across teams—ensures consistent, patient-centred care [60,61]. To achieve the promise of a genuinely unified, patient-focused approach, future implementations should prioritise resolving these systemic coordination and communication gaps.

## 14. Limitations

While this review offers a comprehensive overview of Behavioural Cardiology and presents the HEARTBEAT model as an innovative, integrative framework, several limitations should be acknowledged. First, this review is narrative rather than systematic, which may introduce selection bias and limit reproducibility. Although the literature was thoroughly searched, some relevant studies may have been missed, particularly those in non-English languages or grey literature. Second, the HEARTBEAT model itself has not yet undergone formal, peer-reviewed evaluation, and its outcomes are largely theoretical or based on preliminary institutional data. This limits the strength of recommendations about its scalability and generalisability. Third, while psychosocial risk factors are well-described, the economic, operational, and policy-level barriers to implementation—especially in public healthcare systems—remain underexplored. Finally, comorbid psychiatric conditions such as anxiety, PTSD, and depression are discussed individually, but the interplay between these disorders warrants deeper investigation to guide holistic, real-world application.

## 15. Future Perspectives

As Behavioural Cardiology continues to evolve, several critical challenges and opportunities lie ahead. One of the most pressing issues is the integration of psychosocial assessment into routine cardiovascular care. Despite growing evidence, many cardiology clinics still lack standardised screening protocols, interdisciplinary teams, or referral systems to address psychological distress. Bridging the gap between mental and cardiac care remains a structural challenge, especially in under-resourced settings. Another key hurdle is the scalability of behavioural models like HEARTBEAT, which require dedicated personnel, digital infrastructure, and sustainable funding. Health systems will need to adapt flexible, tiered care pathways that combine in-person and digital interventions. Cost-effectiveness studies, implementation science, and real-world trials are urgently needed to validate these models in diverse populations. In parallel, the growing use of wearable technology, artificial intelligence, and personalised digital coaching offers promising avenues for continuous psychosocial monitoring and early intervention. However, issues of data privacy, digital literacy, and equity must be addressed to ensure that technology bridges gaps rather than deepens them. Finally, future efforts should focus on transdiagnostic approaches that consider the overlapping nature of conditions like depression, anxiety, PTSD, and chronic stress. Understanding how these disorders interact at both behavioural and physiological levels may unlock more effective, patient-centred prevention strategies. In summary, the future of Behavioural Cardiology lies in integrated, scalable, and culturally adaptable models that blend technology, psychology, and cardiology to deliver personalised, equitable care for cardiovascular patients.

## 16. Conclusions

Behavioural Cardiology is emerging as a vital pillar in the prevention, treatment, and rehabilitation of CVD. As the limitations of a purely biomedical model become increasingly apparent, the integration of psychological, social, and behavioural determinants into cardiovascular care is no longer optional—it is essential. This review has demonstrated the profound and multifactorial impact of psychosocial stressors such as depression, anxiety, trauma, socioeconomic adversity, and maladaptive personality traits on cardiovascular health.

The HEARTBEAT model offers a structured, comprehensive, and flexible framework that operationalises these insights into clinical practice. By incorporating holistic assessment, caregiver involvement, validated psychological tools, behavioural interventions, education, team-based care, and technology, HEARTBEAT supports both primary and secondary prevention of CVD. Its adaptability across healthcare settings—from tertiary hospitals to community clinics—makes it a promising strategy for enhancing equity and personalising prevention.

Importantly, the model aligns with current international guidelines and growing global interest in addressing the behavioural determinants of chronic illness. Preliminary data also suggest its potential for improving clinical outcomes and reducing healthcare costs. However, additional research is needed to assess long-term effectiveness, scalability, and cost-efficiency in diverse populations and health systems.

Future directions in Behavioural Cardiology should prioritise the development of culturally sensitive interventions, the integration of digital health tools, and the systematic inclusion of psychosocial screening into standard cardiovascular care. Efforts should also focus on identifying the most effective assessment tools and exploring the precise biological mechanisms linking psychological states to cardiovascular outcomes.

In conclusion, Behavioural Cardiology—through models like HEARTBEAT—represents a forward-thinking, evidence-informed approach that unites cardiology with psychology, public health, and digital innovation. Its implementation has the potential to transform cardiovascular care, reduce global disease burden, and meet the growing need for personalised, human-centred medicine.

## Abbreviations

The following abbreviations are used in this manuscript:

ANS	Autonomic nervous system
CAD	Coronary artery disease
CBT	Cognitive behavioural therapy
CGA	Comprehensive geriatric assessment
CVD	Cardiovascular disease
DASS-21	Depression, Anxiety, and Stress Scales
EQ-5D	EuroQol 5 Dimension
ESC	European Society of Cardiology
GAD-7	Generalized Anxiety Disorder-7
HADS	Hospital Anxiety and Depression Scale
HPA	Hypothalamic–pituitary–adrenal
KCCQ-05	Kansas City Cardiomyopathy Questionnaire—05
LOT-R	Life Orientation Test—Revised
MAFLD	Metabolic dysfunction–associated fatty liver disease
MBSR	Mindfulness-based stress reduction
mHealth	Mobile health
MoCA	Montreal Cognitive Assessment
PHQ-9	Patient Health Questionnaire-9
PSS-10	10-item Perceived Stress Scale
PTSD	Post-Traumatic Stress Disorder
SES	Socioeconomic status
SNS	Sympathetic nervous system
SPQ	Social Problem-Solving Questionnaire
